# Visual attention and memory in professional traders

**DOI:** 10.1038/s41598-023-46905-3

**Published:** 2023-11-16

**Authors:** Francesco Bossi, Andrea P. Malizia, Sonia D’Arcangelo, Francesca Maggi, Nicola Lattanzi, Emiliano Ricciardi

**Affiliations:** 1 MoMiLab Research Unit, IMT School for Advanced Studies Lucca, Piazza San Francesco, 19, 55100 Lucca, Italy; 2 Neuroscience Lab, Intesa Sanpaolo Innovation Center SpA, Torino, Italy; 3 Axes Research Unit, IMT School for Advanced Studies Lucca, Lucca, Italy

**Keywords:** Psychology, Human behaviour

## Abstract

Professional traders need to process a large amount of visual information in their daily activity to judge how risky it is to trade specific investment products. Despite some studies investigating the effects of display clutter on traders, visual attention and memory were never investigated in controlled experimental tasks in this population. Following a preliminary study with 30 participants, visual selective attention and visual working memory were measured and compared between two groups of 15 traders and 15 non-traders (salespeople, acting as a control group) from a large-scale banking group in three experimental tasks measuring selective attention in complex visual contexts, simulating display clutter situations (Visual search), cognitive interference (Stroop task), and a delayed recall visual working memory task. In the Visual search task, traders displayed faster response times (RTs) than non-traders for small display sets, while their performance overlapped for large sets. In the Stroop task, traders showed faster RTs than non-traders but were nevertheless affected by cognitive interference. The memory task highlighted no significant differences between the groups. Therefore, this study found an advantage in traders’ attention when processing visual information in small sets with no retention. This result could influence trading activity—determining an immediate use of relevant visual information in decision making—and traders’ display layout organization.

## Introduction

Professional traders need to process simultaneously an overwhelming amount of visual information in their daily occupation. Indeed, to judge how risky it is to trade a specific investment product, professional traders need to process several pieces of visual information, e.g., price chart, traded volume, 52-weeks high and low, earnings per share, stock volatility, daily price range, competitors’ price change, etc^1^. This information can be organized in customized layouts based on individual traders’ preferences^2^. Nevertheless, excessive visual cues can often lead to attentional phenomena. An example of excessive and redundant visual information can be observed in display clutter^3^. Cluttered display layouts can be an obstacle to optimal decision-making for traders (by increasing the cognitive workload and the frequency of decisional biases) and cause inefficient visual behaviour^4^. Selective attention and, more specifically, choosing relevant information is a crucial factor in successful stock trading^5, 6^, influencing risk preferences in investment choices^7^.

Display clutter is a multifaceted construct that has been broadly studied in ergonomics. Extensively speaking, in display clutters, information density and other properties of task-relevant visualizations are related to effective user performance and visual attention^3^. Visual clutter arises from the interaction among several phenomena, such as high data density, low or weak display organization, and an abundance of irrelevant information. When cluttered, visual information may result in performance and attentional costs related to inefficient use of visual attention. The attentional system plays a crucial role in selecting and processing relevant information from the outside world^8^. In particular, four core processes are fundamental to attention: working memory, top-down sensitivity control, competitive selection, and automatic bottom-up filtering for salient stimuli^9^. Each process contributes to attention distinctly and essentially. Voluntary control of attention involves the first three processes, while bottom-up filtering is the main unintentional component of attention. Meißner & Oll^10^ showed that visual salience and clutter are the most relevant bottom-up processes. In particular, visual salience (related to features like contrast, colour, orientation, and borders) can reliably predict attentional capture^11–13^. Instead, top-down processes are defined as cognitive and goal-directed and are consistently related to task instructions, task-oriented training, practice, and task utility^14–18^.

When operationalizing the display clutter construct, selecting measurement approaches that can capture the various aspects and effects of clutter is fundamental. One of the most relevant and used approaches to study this phenomenon is visual search tasks^3, 19, 20^. In these tasks, the dominant theoretical approach is the *Feature-Integration Theory*^21^. According to this well-supported theory, the visual search process consists of two sequential stages: (i) the first stage is early, preattentive and perceptive, consisting of a fast parallel search of a single target feature; (ii) the second stage is late, attentive and consists of a slower serial search of all objects in the visual scene, aimed at identifying specific conjunction of more than one target features. On the one hand, the so-called *pop-out* effect can often be observed in the first stage (when looking for a single salient feature), i.e., the time needed to identify the target item does not depend on the number of distractors in the visual field. On the other hand, the time needed to identify the target item increases as the number of distractors increases in the second stage (when looking for specific conjunction of features). The parallel search (early stage) is based on the bottom-up processing of stimuli features, while the serial search (second stage) requires a top-down effort to search the visual scene actively. From the display clutter perspective, the performance efficiency in visual search tasks decreases as the display disorder increases.

Another well-documented effect related to selective visual attention is the Stroop effect^22^. The Stroop task is aimed at investigating how the cognitive interference (or cognitive conflict) is processed. The Stroop effect emerges from the interference between the information related to the word vs. the font colour when these are incongruent. Therefore, selective attention is needed to select the relevant information to the task while ignoring irrelevant information. For this reason, the Stroop effect is often explained by a conflict between top-down (i.e., task instructions) and bottom-up processing (i.e., the salience of the written word)^23^. Consequently, the Stroop task can be a decisive task to investigate visual selective attention in traders in relation to display clutter phenomena. Although this task is distant from a typical visual search task (particularly with respect to spatial aspects of attention), it is fundamental to study how visual information is selected and processed while excluding irrelevant information.

A final aspect strictly related to visual attention is visual working memory^9^. The construct of working memory has undergone several definitions and conceptualizations. Still, it can be broadly defined as the retention of a small amount of information that can be held in mind in a readily accessible buffer and used to execute cognitive tasks^24^. Visual working memory is considered to be one of the core components of visual attention, along with the interaction between top-down and bottom-up attentional processes^10^; visual attention filters incoming information, allowing only relevant information into short-term processing stores but, vice-versa, visual working memory can also influence the guidance of selective attention^25^. Accordingly, investigating visual working memory in traders is crucial to understanding how they retain and process visual information to guide decision-making related to stock trading.

### Aim of the study

Despite their relevance in processing visual information concerning display clutter, to our knowledge, these processes were studied in traders only by using Direct Access Trading platforms for real stock market negotiations^4, 26^. These studies highlighted decisive aspects of decision-making processes in traders (especially related to expertise) but could not draw general conclusions about selective attention mechanisms in this population. Experimentally controlled tasks are generalizations of everyday tasks for professional traders and allow studying specific components of selective attention in a more controlled paradigm. Since we already have some insights about how traders handle display clutter in the context of their everyday tasks, it is crucial to determine how these cognitive processes extend and generalize to visual search and selective attention tasks, and compare traders to a non-trader population. This is a decisive aspect since using experimental tasks allows comparing traders to non-traders, thus understanding how the trader population differs from the general population in these facets. A comparable and adequate control group is fundamental for identifying the peculiarities of traders. Investigating visual attention by using trading platforms has the advantage of being more ecological, but the critical disadvantage of not allowing a direct comparison between traders and non-traders.

Given the limited previous literature on the topic^4^, we expected traders to be more efficient than non-traders in visual attention tasks. From this premise, we hypothesized (i) better performance for traders in the Visual search task, i.e., higher accuracy, faster response times, and lower difference between conjunction search vs. feature search conditions; (ii) better processing of cognitive interference for traders in the Stroop task, i.e., higher accuracy, faster response times, and lower difference between incongruent vs. neutral and congruent conditions; (iii) better performance for traders in the visual working memory task, i.e., better recall scores.

## Results

### Preliminary results

In a preliminary study with 30 participants out of the bank context, we based our preliminary analysis on the results from the Stroop task, since it showed the highest statistical robustness in effect size estimation^27, 28^ among the tasks we used in our protocol. A linear mixed-effects model showed a statistically significant main effect of congruity (*F*_(2, 58)_ = 37.079, *p* < 0.001). Post-hoc comparisons (corrected according to Tukey’s HSD) revealed that incongruent trials showed significantly higher response times than both congruent (*t*_(58)_ = 7.553, *p* < 0.001) and neutral trials (*t*_(58)_ = 7.359, *p* < 0.001), while the latter two were not significantly different (*t*_(58)_ = -0.194, *p* = 0.980). We based our Cohen’s *d* estimation on the lowest t-value in the comparisons between the incongruent condition and any other condition (i.e., *t* = 7.359) to have a conservative estimate. By setting *n* = 30, we obtained *d* = 1.344 (corresponding to a very large effect size). Based on this effect size, we performed a power analysis for two-sample tests, with α significance level = 0.05 and power = 0.95, with a two-sided alternative hypothesis. Therefore, since we obtained *n* = 15.4, we selected a sample size of 15 participants per group in the main study.

### Visual search task

**Figure 1:**
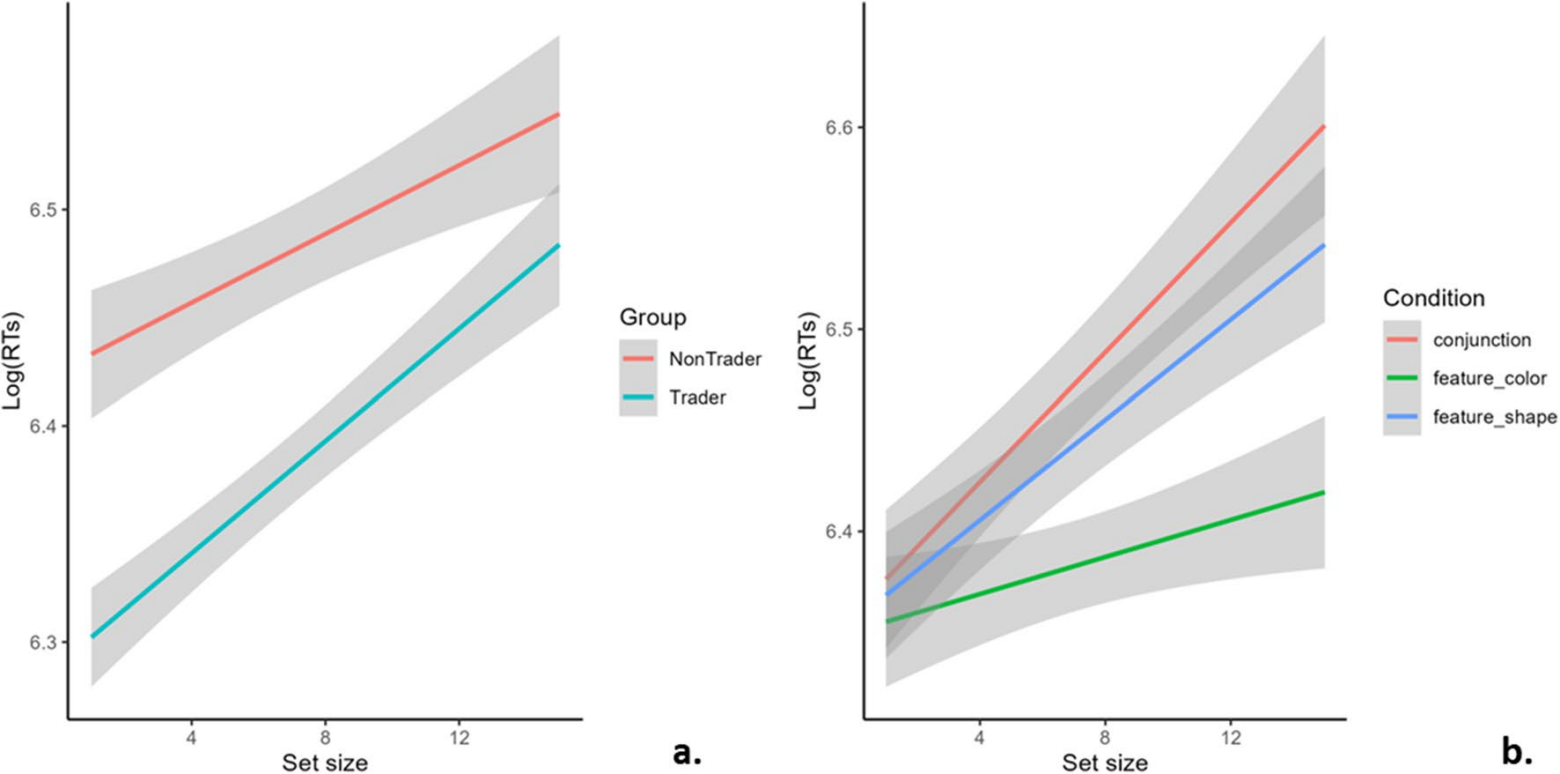
Visual search results. This figure represents the main results from the Visual search task. In both panels, the x-axis represents the set size (i.e., the number of figures in the display set, ranging from 1 to 15), while the y-axis represents participants’ log-transformed response times (RTs), predicted regression lines are represented and the shaded area represents 95% confidence interval. Panel A represents the group * set size effect: traders showed lower RTs than non-traders in small display sets, their regression line increased with a significantly steeper slope, and RTs from the two groups overlapped in large display sets. Panel B represents the condition *Set size effect: RTs increased with a smaller (yet significant) slope in the feature-colour condition than in the feature-shape and conjunction conditions.

In the Visual search task, the analyses of the accuracy did not bring conclusive results since all effects were negligible (all *Fs* < 0.6). This is most probably due to the extremely low number of incorrect trials (i.e., 15/1620). In the RTs model, the main effect of set size (*b* = 0.008, *F*_(1, 1490.11)_ = 184.1, *p* < 0.001) and the group * set size (*F*_(1, 1490.11)_ = 11.32, *p* < 0.001) and condition * set size (*F*_(2, 1490.10)_ = 15.72, *p* < 0.001) interaction effects resulted as statistically significant. The condition * group * set size three-way interaction did not result as statistically significant (*F*_(2, 1490.10)_ = 0.723, *p* = 0.486). The set size positive main effect showed that RTs increased as the set size increased. The simple slope analysis on the group * set size interaction effect displayed a positive significant effect for both groups (traders: *b* = 0.013, *t*_(1490)_ = 11.984, *p* < 0.001; non-traders: *b* = 0.008, *t*_(1490)_ = 7.210, *p* < 0.001), but with a significantly greater slope in traders than non-traders (Fig. 1a). In particular, RTs were faster for traders compared to non-traders in low set size values, while they tended to overlap for higher set size values. The simple slope analysis performed on the condition * set size interaction effect showed that the set size effect on RTs was statistically significant in all conditions, but with significantly different slopes: feature search-colour condition: *b* = 0.005, *t*_(1490)_ = 3.592, *p* < 0.001; feature search-shape: *b* = 0.012, *t*_(1490)_ = 8.985, *p* < 0.001; conjunction search: *b* = 0.015, *t*_(1490)_ = 10.688, *p* < 0.001 (Fig. 1b).

### Stroop task

In the Stroop task analysis, the accuracy analyses highlighted the two main effects (group: *F*_(1)_ = 3.061; congruity: *F*_(2)_ = 2.476), while the 2-way interaction effect was negligible (i.e., *F*_(2)_ = 0.744). Statistical significance was computed with likelihood ratio tests (LRTs) via models comparison. While the group main effect was not significant (χ^2^_(1)_ = 1.948, *p* = 0.163), the congruity main effect proved to be statistically significant (χ^2^_(2)_ = 6.344, *p* = 0.042). However, Tukey-corrected post-hoc comparisons showed no significant comparisons between congruity conditions: all *z*s < 1.8, all *p*s > 0.17. As in the Visual search task, these results are most probably due to the extremely low number of incorrect trials (i.e., 25/2160).

**Figure 2:**
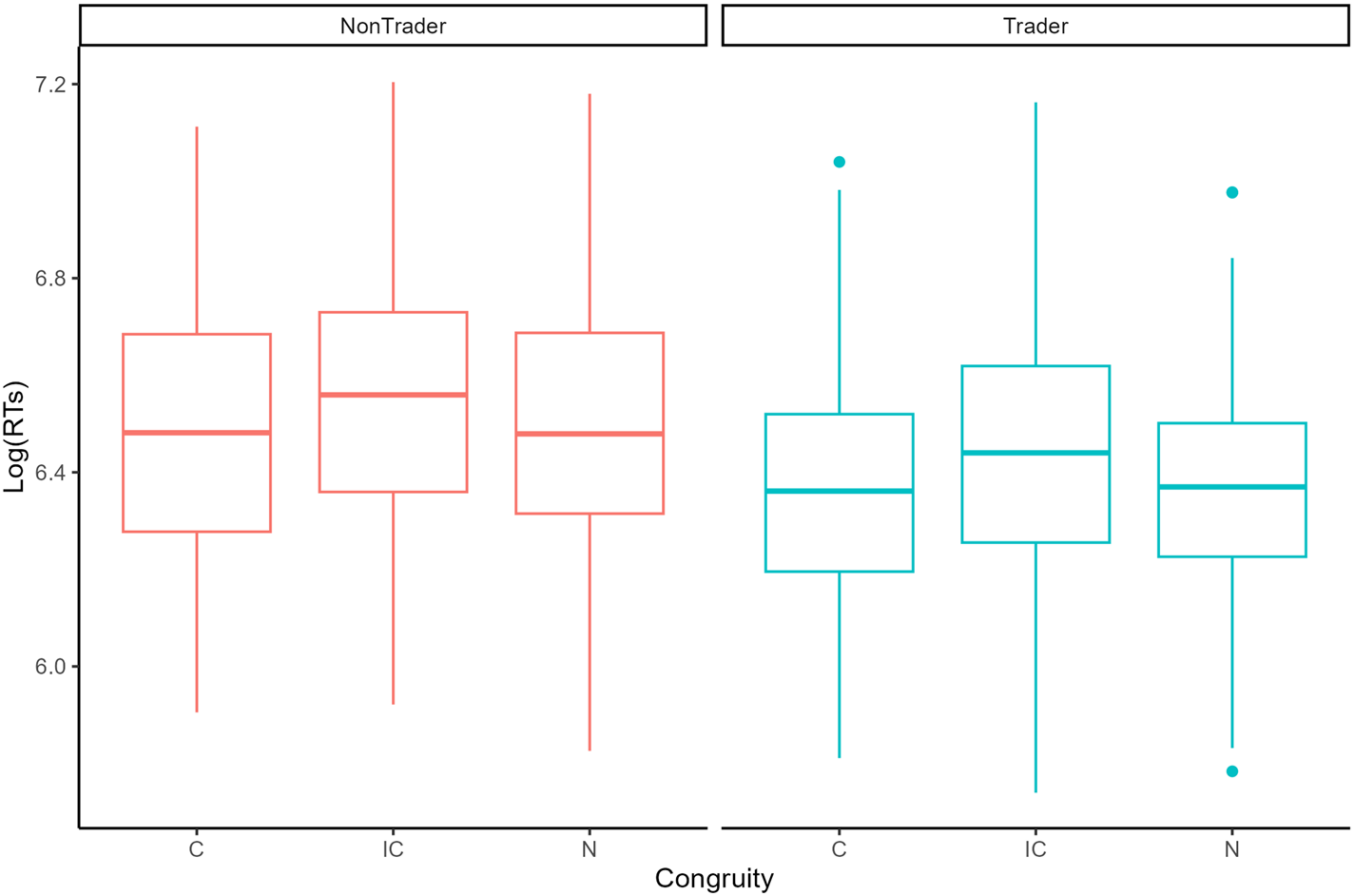
Stroop task results. This figure shows the boxplot of participants’ log-transformed response times (RTs, on the y-axis), in two separate groups (traders vs. non-traders). The x-axis represents the Stroop congruity condition, i.e., *C* congruent, *IC* incongruent, *N* neutral. The boxplot shows overall faster RTs for traders compared to non-traders. The cognitive interference effect (i.e., slower RTs for incongruent compared to congruent and neutral conditions) is present in both groups.

In the RTs model, the main effects of group (*F*_(1, 28)_ = 4.703, *p* = 0.039) and congruity (*F*_(2, 2012.1)_ = 29.32, *p* < 0.001) were statistically significant (Fig. 2). The group effect showed that traders (mean = 611 ms) displayed faster RTs than non-traders (mean = 695 ms), revealing thus greater efficiency in the task. The congruity effect showed that incongruent trials (mean = 688 ms) presented significantly slower RTs than congruent (mean = 633 ms; *t*_(2012)_ = 7.003, *p* < 0.001) and neutral ones (mean = 639 ms; *t*_(2012)_ = 6.257, *p* < 0.001), which did not differ significantly (*t*_(2012)_ = 0.784, *p* = 0.713).

The group * congruity interaction effect did not result as statistically significant (*F*_(2, 2012.1)_ = 0.957, *p* = 0.384). Therefore, the null hypothesis (i.e., both groups were affected by cognitive interference in the same way) cannot be rejected.

### Visual working memory recall task

The analysis of the Visual working memory recall task showed no statistically significant differences in the recall score between traders and non-traders (*F*_(1, 28)_ = 3.014, *p* = 0.094). Traders showed a lower mean recall score (9.47) than non-traders (12.13), but this difference was not statistically significant.

## Discussion

Professional traders have to process a great amount of visual information in their daily occupations. In the present study, visual attention and memory were investigated and compared between traders and non-traders in a Visual search task, a Stroop task, and a delayed recall visual working memory task. The Visual search task showed that traders were more efficient than non-traders when processing a low number of stimuli (i.e., low set size values), while performance in the two groups tended to overlap for the higher number of stimuli (i.e., RTs increased more as the number of distractors in the display set increased). In the Stroop task, traders displayed greater efficiency in the task (i.e., faster RTs) than non-traders in processing relevant information. Still, both groups were similarly affected by cognitive interference. The visual working memory task highlighted no significant differences between traders and non-traders.

Some relevant studies investigating the phenomenon of display clutter in the ecological context of real or simulated stock market negotiations have been previously performed. The study from Ognjanovic and colleagues^4^ showed that novice traders are more affected by high display clutter than experts on both performance and visual attention measures; moreover, evidence from a neuroimaging study^26^ showed that trading is handled as a well-learned automatic behaviour by expert traders, while it needs more attentional effort from novice traders. Nevertheless, visual attention and memory were never investigated before by means of controlled experimental tasks in traders, comparing their performance to a non-trader population. Indeed, this represents the main innovation of our study.

In the Visual search task, the condition * set size interaction effect demonstrates the Feature-Integration Theory, based on which the Visual search task was created. Indeed, the number of distractors affects participants’ performance to a greater extent during serial search (i.e., in the conjunction condition) as a result of top-down effort and less during parallel search (i.e., feature-colour and feature-shape conditions), in which bottom-up processing is involved. In these two latter conditions, the set size significantly affected participants’ performance, but not as much as the conjunction condition. This result reveals the absence of a *pop-out* effect, probably related to the salience of the stimuli’ shape and colour^11, 12, 29, 30^.

Moreover, the group * set size interaction effect showed that traders were overall less affected by distractors when compared to non-traders, with small set sizes. At the same time, their performance tended to overlap with large set sizes. Therefore, traders were more efficient in processing small sets of figures. Still, their performance worsened more as the number of distractors increased (as represented by traders’ steeper slope in Fig. 2a), resulting in a comparable performance in large sets for traders and non-traders. This shows that traders tend to be more efficient than non-traders in processing visual information but are nevertheless affected by phenomena such as visual clutter. This result partly confirms our first hypothesis. However, it is crucial to highlight that this effect occurs regardless of the Visual search condition, as the three-way interaction is not statistically significant. Accordingly, both traders and non-traders appear to use serial vs. parallel search according to the experimental condition. This result implies the preference toward simplified layouts in trading platforms: these layouts need to provide all the information that is indispensable to deciding about trading stock, but nothing more. Indeed, Ognjanovic and colleagues^4^ proved that cluttered displays can directly affect financial risk judgment, especially in novice traders. This kind of layout would allow efficient and fast processing of relevant information since traders show to be affected by visual clutter as much as non-traders, despite their general advantage with small sets.

Stroop task results are analogous to those observed in the Visual search task. The main effect of congruity confirmed the proper Stroop effect as known in the classic literature^22^, i.e., irrelevant text information is more difficult to ignore in the incongruent condition than in congruent and neutral conditions. This result confirms the cognitive interference effect, as formulated by Stroop and confirmed by further literature^23^. In the main effect of group, traders displayed overall greater efficiency than non-traders in processing relevant information. This result partly confirms our second hypothesis by proving that traders are more efficient than non-traders in the Stroop task, i.e., processing relevant visual information while excluding irrelevant information. Since previous literature has shown that more expert traders tend to be less affected by display clutter effects^4^, this result could be related to traders’ everyday occupations. Having to process a great amount of visual information daily, traders could have developed more efficient selective attention skills compared to their non-trader colleagues, thanks to their experience. The same interpretation also applies to the Visual search results. However, these results could be potentially interpreted in the opposite direction: people displaying better selective attention in small sets may be more inclined to choose a career in trading relative to other jobs in the banking sector. These two interpretations could be disambiguated in a future study considering the effects of experience in trading (i.e., job seniority).

Similar to the results found in the Visual search task, the absence of a significant group * congruity interaction effect proved that the cognitive interference affected their visual information processing as much as it did in non-traders. This result indicates that traders are still prone to cognitive interference from incongruent visual information, generated by a conflict between top-down and bottom-up processing, despite being more efficient in processing this kind of information overall. Translating this result in the context of a trading platform implies that traders are more efficient in interpreting the relevant information necessary for trading decisions. However, they are still prone to display clutter effects, i.e., irrelevant information on the screen can still interfere with their decision-making.

Finally, no significant differences were observed between traders and non-traders in the delayed recall visual working memory task. This suggests that traders show some specific advantages in processing visual information in the immediate moment (as they could benefit from this advantage in their occupation); still, they do not retain this kind of information longer than their non-trader colleagues. In the context of professional trading, this difference between immediate vs. delayed use of visual information could imply that traders tend to complete their decision-making processes immediately after processing relevant information without delaying their decision. However, we cannot exclude that the difference in recall scores between traders and non-traders was not statistically significant just because of the small sample sizes. Therefore, it is risky to make speculative interpretations about this null result.

The main limitation of this study is the relatively small sample size. Unfortunately, due to COVID-19 restrictions and limited availability of bank employees, it was possible to recruit only thirty participants working in a banking group who were available for in-person testing. Nevertheless, the choice of this sample size is supported by our preliminary results, which showed sufficient power. Moreover, the two groups of participants were balanced for age and gender, leading to more comparable results.

## Conclusions

In order to investigate visual attention and memory in stock traders and non-traders, we compared their performance in a Visual search task, a Stroop task, and a delayed recall visual working memory task. We found that traders were more efficient than non-traders in processing relevant visual information, particularly with small display sets of items. Nevertheless, they were subject to the effects of serial search and cognitive interference as much as non-traders and did not display any differences in visual memory. Accordingly, traders showed an advantage in processing visual information in small sets without retention, which could play a relevant role in their occupation. This is a seminal study to investigate visual attention in professional traders, not only in ecological trading platforms but also in general tasks allowing the comparison of cognitive processes with non-traders. Future studies stemming from this line of research could investigate in further detail how the processing of visual information is related to decision-making (thus to the use of heuristics and biases) and how these processes are identified by specific neurofunctional markers in the nervous system.

## Methods

### Participants

Thirty healthy employees in a large-scale banking group were recruited for this study. Fifteen of these participants (2 F, mean ± sd age: 39.5 ± 8.9) were professional investors and carried out this activity daily. The remaining 15 participants (2 F, mean ± sd age: 40.7 ± 8.9) were employed as salespeople in the same banking group. All participants had been carrying out their occupation in the banking group for at least one year at the moment of data collection. All participants had normal or corrected-to-normal vision and did not report colour blindness. The sample size was identified thanks to a power analysis performed on a preliminary study (see Sect. “Preliminary study”).

### Ethical statement

All participants were provided with an exhaustive description of all the experimental procedures and were required to sign a written informed consent before taking part in the study. The study was conducted in accordance with the ethical standards laid down in the 1964 Declaration of Helsinki and under a protocol approved by the Area Vasta Nord Ovest Ethics Committee (protocol n. 24579/2018).

### Materials and procedure

**Figure 3:**
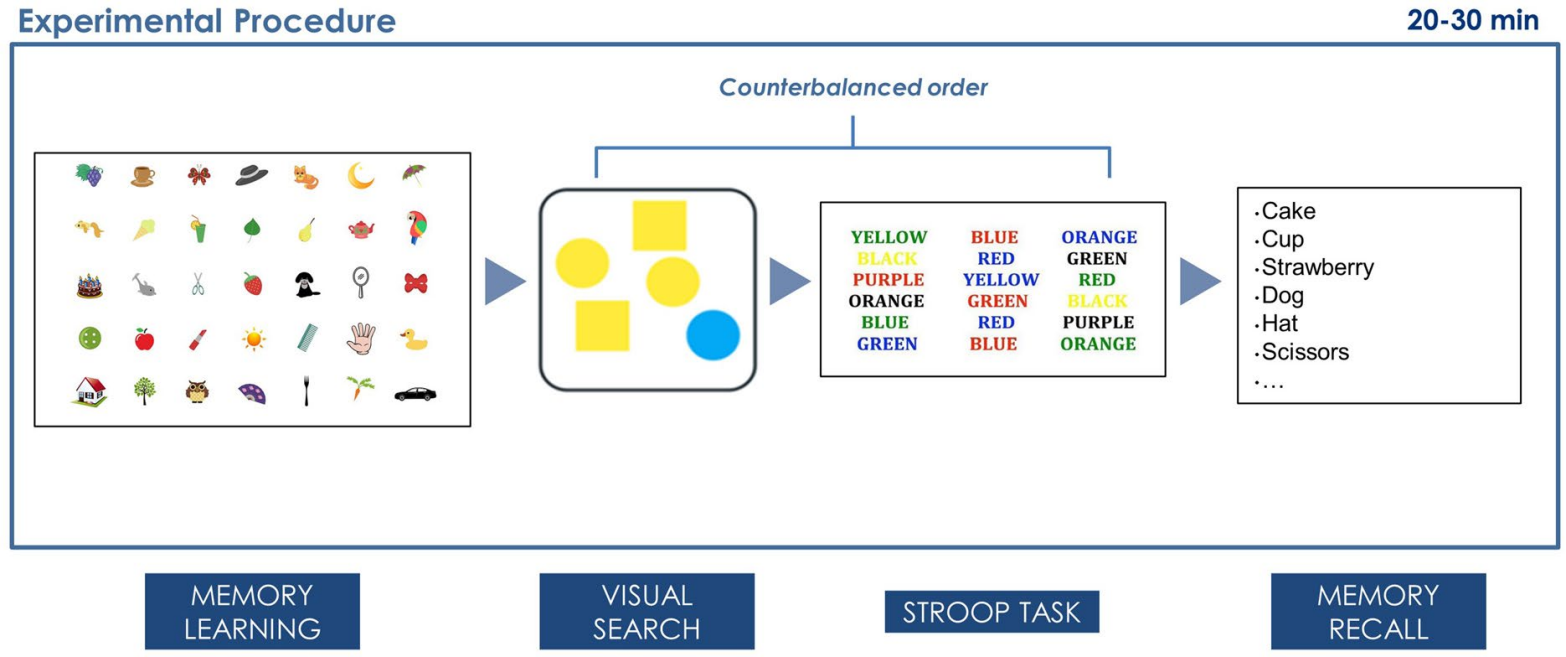
Experimental procedure. This figure depicts the experimental procedure of this study. In the first phase (learning phase), participants were asked to memorize the items in the picture for one minute. Then, in a counterbalanced order across participants, they performed the Visual search task and the Stoop task. Finally, they were asked to write down all the items they remembered from the learning phase, in any order (delayed recall visual working memory task). The typical duration of the whole procedure was 20 to 30 min.

The experiment was carried out in a dimly illuminated room with constant internal illumination. After reading and signing the informed consent, participants were seated 100 cm from a 21-inch LCD monitor. All tasks were presented on OpenSesame version 3.1^31^. The whole experiment duration was approximately 20–30 min. The experimental procedure is outlined in Fig. 3.

**Figure 4:**
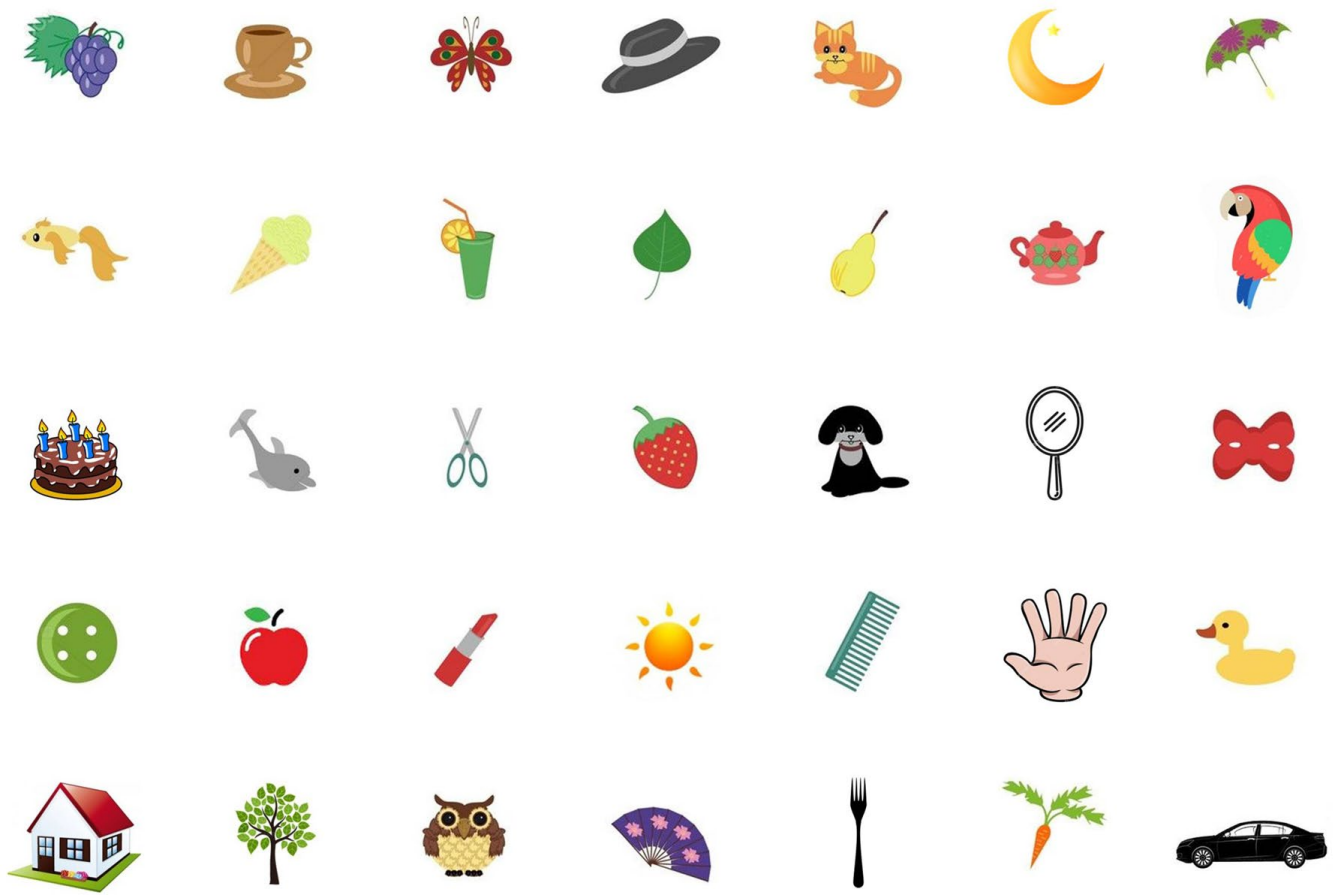
Visual working memory task. This figure reproduces the items display shown to participants for one minute during the learning phase. This figure portrays 35 items of most frequent use in daily life or common animals.

Participants were first requested to perform the learning phase of the visual working memory task. In this phase, participants were requested to look at the picture depicted in Fig. 4, shown on the screen for one minute. Their task was to memorize as many items as possible. Instructions explicitly stated the need to perform a delayed free recall (in no particular order) of these items. Still, they gave no specific suggestions about any strategies to be used to learn those items. Items in the picture were selected among items of most frequent use in daily life or common animals.

After the learning phase, participants were asked to perform two experimental tasks, presented in a counterbalanced order across participants: the Visual search task and the Stroop task.

The Visual search task consisted of a revisited version of the traditional visual search task by Treisman & Gelade (1980). It was aimed at measuring visual selective attention based on the Feature Integration Theory (*ibidem*). This task consisted of four experimental blocks presented in a random order, each with a different target figure varying in shape and colour (i.e., yellow square, blue square, yellow circle, blue circle). Specific instructions were shown at the beginning of each block. Each block included 18 trials, presented in random order. In each trial, a display of randomly positioned figures was shown after 500 ms of the fixation cross and until response. Participants were instructed to press the left arrow key (marked with a “NO” label) as fast and accurately as possible if they could not find the target figure in the display, or the right arrow key (marked with a “YES” label) if they found the target figure. Feedback about the response correctness was given after each trial for 500 ms before the following trial started. General feedback about average response times and accuracy was given at the end of each block.

The target figure was present in half of the displays and absent in the remaining half. Each trial in the block varied according to two variables: set size, i.e., each display could consist of 1, 5, or 15 figures randomly positioned on the screen; and condition, i.e., one of the three experimental conditions (feature search-colour, feature search-shape, or conjunction search). In the feature search-colour condition, all distractors presented a different colour from the target colour (e.g., blue circle target among yellow circle and square distractors). In the feature search-shape condition, all distractors presented a different shape from the target colour (e.g., blue circle target among yellow and blue square distractors). In the conjunction condition, both shape and colour differed between the target and distractors (e.g., blue circle target among blue square, yellow square, and yellow circle distractors). According to the Feature Integration Theory, a greater worsening of participants’ performance is expected as the set size increases in the conjunction condition (due to the serial search process, which is less efficient and less accurate) compared to the feature conditions (in which the more efficient parallel search is required). This task’s duration was approximately 5–10 min.

The Stroop task consisted of a behavioural PC transposition of the classic Stroop task^22^. This task was used to test the cognitive interference processing skills. The task was divided into a practice phase consisting of 18 trials and a test phase consisting of 72 trials, divided into two experimental blocks of 36 trials each, with a pause screen between the two blocks. Trials were presented in a random order in both phases. Instructions were presented at the experiment start and later summarized after the practice phase. Feedback was given at the end of the practice phase, reporting the participant's average response times (RTs) and accuracy. Each trial consisted of a black screen presented for 1,000 ms, followed by a fixation cross in the centre of the screen for 200 ms. As the fixation cross disappeared, the target word was presented in the centre of the screen until response. Participants were instructed to press a key on the keyboard as fast and accurately as possible based on the font colour of the target word: three keys were marked with three coloured labels, i.e., red on the V key, green on the B key and yellow on the N key. After the participant’s response, the following trial started. Target words were equally distributed among the three colours and three conditions: congruent (the font colour matched the target word, e.g., “GREEN” written in green), incongruent (the font colour did not match the target word and was one of the two remaining colours, e.g., “GREEN” written in red), and neutral (the target word was a string of Xs, e.g., “XXX” written in green). Expected cognitive interference processing skills were measured by the performance difference between the incongruent condition vs. congruent and neutral conditions. This task’s duration was approximately 5–10 min.

After these experimental tasks, the study was concluded by the free recall phase of the visual working memory task. During this phase, participants had 3 min to write in a text input window all the items they remembered from the picture shown at the beginning of the experiment, in no particular order. Participants were then debriefed about the experiment and dismissed.

### Preliminary study

Given the COVID-19 restrictions and the limited availability of bank employees, we performed a preliminary study in order to identify the ideal number of participants to obtain a reliable statistical power. To pursue this goal, we administered the same procedure to 30 healthy volunteers (11 F, mean ± sd age: 27.8 ± 4.4) out of the bank context. We then derived Cohen’s *d* as effect size measure and performed a statistical power analysis to obtain *n*, i.e., the minimum number of participants per group in the main study.

### Statistical analyses

In order to obtain more reliable measures and in line with the previous literature^32^, accuracy and RTs were analysed separately on a single trial basis in the Visual search and Stroop tasks. Response times were cleaned by removing incorrect trials and outliers above and under 2 st. dev. from the individual mean, for each participant in each condition, i.e., 9 conditions in the Visual search task (set size = 1, 5 or 15; feature search-colour, feature search-shape, or conjunction search) and 3 conditions in the Stroop task (congruent, incongruent and neutral). Log-transformed RTs were used instead of simple RTs to increase the normality of the distribution. The number of correct items written in the free recall phase was used as participants’ scores in the visual working memory task. Only correct items were counted in the final score, and penalties were not computed for incorrect items recalled.

In the Visual search task, accuracy and RTs were analysed by using a random-intercept mixed model (generalized mixed model on a binomial distribution for accuracy) to account for individual variability^33, 34^, with group (2-level factor: traders vs. non-traders), set size (continuous, ranging from 1 to 15) and congruity (3-level factor: feature search-colour vs. feature search-shape vs. conjunction search) as independent variables in interaction. In the accuracy model, the full factorial model could not converge. Therefore only main effects and the group * set size two-way interaction were included as predictors since any other models could not converge. Accuracy and RTs in the Stroop task were similarly analysed by using a random-intercept mixed model (generalized mixed model on a binomial distribution for accuracy) with group and congruity (3-level factor: congruent vs. incongruent vs. neutral) as independent variables in interaction. Memory score was analysed by using a linear model with the group as independent variable. Post-hoc pairwise comparisons’ and simple slope analyses’ p-values were corrected according to Tukey’s HSD correction. All degrees of freedom in mixed models are computed using the Satterthwaite method.

All statistical analyses were performed in RStudio software^35^. Mixed-effects models were estimated by using the *lme4* and *lmerTest* packages^36, 37^; simple slope analyses and post-hoc pairwise comparisons were performed with the *gamlj* package^38^, based on *emmeans*^39^; plots were created using the *ggplot2* package^40^; the power analysis was performed using the *pwr* package^41^.

## Data Availability

Data and materials are currently available via the following link: https://osf.io/duxpr/
